# Machine learning driven LD_50_ prediction for cancer risk assessment using modern molecular language models

**DOI:** 10.3389/fonc.2026.1788240

**Published:** 2026-03-19

**Authors:** Tanuj Sharma, Peter Sona, Jongsun Jung

**Affiliations:** AI Drug Discovery and Development, Syntekabio, Inc., Daejeon, Republic of Korea

**Keywords:** ChemModernBERT, LD50 prediction, LLM, oral toxicity, toxicity prediction

## Abstract

**Introduction:**

Accurate assessment of chemical toxicity is fundamental to cancer research, where early identification of hazardous compounds is critical for prioritizing carcinogenicity testing, therapeutic safety evaluation, and regulatory decision-making.

**Methods:**

We developed ChemModernBERT, a ModernBERT-based molecular language model pretrained using a curriculum learning strategy on more than 1.8 million SMILES strings to generate chemically informed sequence representations. Using a curated dataset of 8,898 compounds, we systematically compared four molecular representation learning approaches ChemBERT, ChemProp (a directed message passing neural network), ensemble learning, and ChemModernBERT for predicting median lethal dose (LD_50_) values. Model performance was evaluated using standardized internal and external test sets.

**Results:**

ChemModernBERT achieved the lowest mean absolute error (MAE) on both internal (0.390) and external (0.393) evaluations and the highest coefficient of determination (R^2^ = 0.521 on the external test set), outperforming ChemBERT, ChemProp, and ensemble models under identical experimental conditions. The small generalization gap between internal and external evaluations indicates strong transferability across chemically diverse compounds.

**Discussion:**

These findings demonstrate that curriculum-pretrained transformer architectures provide a scalable and accurate framework for large-scale toxicity prediction. Such models can support computational pipelines for carcinogenicity assessment, dose selection, and early-stage chemical safety evaluation.

## Introduction

Toxicological assessment is a cornerstone of chemical safety evaluation, underpinning regulatory decision-making in pharmaceuticals, environmental health, and industrial chemistry. Among the most widely used quantitative measures of acute toxicity is the median lethal dose (LD_50_), defined as the dose required to cause mortality in 50% of a test population and typically expressed in milligrams of substance per kilogram of body weight (1–3). LD_50_ values provide a standardized and interpretable metric for comparing the short-term toxic effects of chemical compounds and are routinely employed by regulatory agencies to support hazard classification, exposure limit setting, and risk communication (4–6). Despite its broad regulatory utility, LD_50_ captures only acute lethality and does not reflect the full spectrum of adverse health outcomes associated with chemical exposure. In particular, chronic toxicological effects most notably carcinogenesis are not adequately represented by acute toxicity metrics alone (7). Cancer, a leading cause of morbidity and mortality worldwide, often arises from prolonged or repeated exposure to chemical agents that induce genomic instability, disrupt cellular signaling pathways, or promote aberrant cell proliferation (8, 9). Such carcinogenic effects may manifest over extended latency periods, frequently in the absence of overt acute toxicity. Consequently, compounds with relatively low acute lethality may still pose substantial long-term cancer risks, especially under conditions of sustained exposure. This disconnect highlights a fundamental limitation of relying exclusively on LD_50_ values for comprehensive chemical safety assessment and underscores the need for integrative computational frameworks that can support both acute and chronic toxicity evaluation. This is particularly relevant for carcinogenicity prioritization, where early toxicity estimation informs compound selection.

Traditional experimental approaches for toxicity assessment, including *in vivo* LD_50_ determination and long-term carcinogenicity studies, are resource-intensive, ethically constrained, and impractical for large-scale chemical screening (10–12). To address these limitations, computational approaches particularly machine learning (ML)—have emerged as powerful tools within modern toxicology workflows (13–20). Conventional ML-based LD_50_ prediction models often depend on handcrafted molecular descriptors or predefined fingerprints, which may encode prior assumptions, limit representational flexibility, and hinder generalization across structurally diverse chemical spaces. Recent advances in large-scale molecular representation learning have transformed this landscape (21). In particular, transformer-based language models trained on chemical string representations such as Simplified Molecular Input Line Entry System (SMILES) have demonstrated strong capability for capturing complex structural patterns directly from raw molecular sequences (22–26). By treating molecular sequences as a formal language, transformer-based architectures can learn rich, contextualized representations that capture substructural patterns, stereochemical relationships, and long-range dependencies that are difficult to encode using traditional descriptor-based methods (27–29).

Current LD_50_ prediction methods largely optimize predictive accuracy but do not explicitly evaluate representation transferability or scalability across diverse chemical domains. Recent advances in molecular language modeling (30) have further demonstrated that the quality of pretrained representations critically depends on both dataset scale and training strategy. In particular, curriculum-based pretraining where models are first exposed to canonical SMILES before being progressively trained on more diverse and structurally complex representations has been shown to improve training stability, syntactic robustness, and downstream generalization (29, 30). Building on this paradigm, we employ a ModernBERT architecture pretrained on large-scale SMILES corpora, providing a strong and transferable molecular representation backbone for toxicity prediction tasks. In this study, we leverage modern SMILES-based transformer architectures to develop high-performance regression models for LD_50_ prediction, providing a scalable and data-efficient alternative to conventional toxicity modeling pipelines. Beyond improved predictive accuracy, these models offer a flexible foundation for extension to additional toxicological endpoints, including carcinogenicity and other chronic health outcomes relevant to cancer risk assessment. By integrating acute toxicity prediction with emerging molecular language modeling approaches, this work contributes to the development of computational frameworks capable of supporting early-stage hazard identification, prioritization of compounds for carcinogenicity testing, and reduction of animal testing burdens. This might assist in carcinogen prediction as early toxicity estimation help to select compounds. Such advances are particularly relevant to cancer research and regulatory toxicology, where rapid and reliable assessment of chemical hazards is essential for protecting human health and informing evidence-based decision-making.

## Materials and methods

### Dataset collection and preprocessing

Experimental LD_50_ values were obtained from publicly available chemical and toxicological databases maintained by the U.S. National Library of Medicine and the U.S. Environmental Protection Agency (31). Only experimentally measured acute oral LD_50_ records expressed in milligrams per kilogram (mg/kg) and associated with valid chemical structures were retained. Each entry comprised a chemical identifier, molecular structure encoded as a SMILES string, LD_50_ value, measurement unit, and relevant metadata. Records containing missing values, malformed or unparseable SMILES strings, or non-numeric toxicity measurements were excluded. To ensure consistency and eliminate redundant representations, all SMILES strings were canonicalized using RDKit (32), enforcing a unique string representation for each molecular graph and preventing duplication arising from alternative SMILES encodings; molecules that failed canonicalization were removed. A detailed preprocessing script specifying each sanitization and standardization step has been described in supplementary section S1. LD_50_ values were transformed to a logarithmic scale (log_10_ LD_50_) to reduce skewness, stabilize variance, and improve numerical stability during model training. When multiple experimental LD_50_ measurements were available for the same compound, the values were aggregated into a single target using a robust statistical procedure designed to mitigate the influence of experimental variability and outliers. The TDC dataset was included only for descriptive comparison of dataset characteristics and was not used for model training, validation, or benchmarking to avoid confounding effects arising from heterogeneous annotation standards and endpoint definitions.

### Pretraining of the ModernBERT SMILES model

The ModernBERT SMILES model (33) was pretrained on approximately 1.8 million unique SMILES strings curated from the ChEMBL database. All molecules were processed using a defined preprocessing pipeline implemented in RDKit. *Sanitization* refers to RDKit’s structure validation procedures, including valence checks, aromaticity assignment, explicit/implicit hydrogen normalization, and removal of chemically invalid structures. *Standardization* was performed separately and consisted of salt stripping, charge normalization, functional group normalization, tautomer standardization, and canonical SMILES generation. Canonicalization was therefore only one component of the broader standardization protocol. Molecules that failed sanitization were excluded prior to training to ensure chemical validity and dataset consistency. Pretraining followed a two-stage curriculum learning paradigm designed to progressively increase the structural complexity of input sequences. In the first stage, the model was trained for 20 epochs on ~1.1 million canonical SMILES, enabling stable learning of core SMILES grammar, atom-level syntax, and common functional group patterns. In the second stage, training continued for 10 epochs on an expanded corpus of ~1.8 million extended SMILES representations containing alternative atom orderings of the same molecular graphs. This stage enhanced robustness to representational variability and improved generalization across diverse chemical encodings. The model was trained using a masked language modeling (MLM) objective (34), where a fixed proportion of input tokens were randomly masked and the model was optimized to predict the original tokens based on surrounding context. The masked language modeling objective was selected because it encourages bidirectional contextual reasoning over molecular tokens, enabling the model to infer chemically valid substructures from partial information. Unlike autoregressive objectives, MLM promotes global structural awareness, which is particularly important for molecular property prediction where toxicity often depends on distributed functional group interactions rather than local motifs alone. Optimization was performed using the AdamW optimizer (35) with standard transformer training settings. The final pretrained model was saved in model.safetensors format along with the associated tokenizer configuration. This curriculum design was motivated by the hypothesis that staged exposure to standardized followed by structurally diverse representations enables molecular language models to first learn stable syntax statistics and then acquire graph-level invariances. Such representations are expected to generalize more reliably across chemically heterogeneous compounds, which is essential for downstream predictive tasks such as toxicity estimation and cancer-risk prioritization.

### Model training for LD_50_ prediction

Each molecule was represented as a canonical SMILES string and tokenized using the same subword tokenizer employed during pretraining. This ensured full consistency between pretraining and downstream fine-tuning. The tokenizer decomposes SMILES strings into chemically meaningful tokens, including atoms, bonds, ring indices, and branching symbols. Token sequences were padded or truncated to a fixed maximum length and augmented with rotational positional embeddings. The prediction model is based on an encoder-only transformer architecture (ModernBERT) specialized for molecular sequences (36). The encoder consists of a stack of identical transformer layers, each comprising of Multi-head self-attention mechanisms to capture both local and long-range dependencies within the molecular sequence. Position-wise feed-forward networks with nonlinear activation function GELU was used. Residual connections and layer normalization were used to stabilize training and improve gradient flow. Through self-attention, the model learns contextualized token embeddings that encode both SMILES syntax and implicit chemical structure, including functional group interactions and scaffold-level relationships. To obtain a fixed-dimensional molecular embedding suitable for regression, a pooling operation was applied to the final hidden states of the transformer. In the default configuration, the embedding corresponding to the special [CLS] token was used as a global molecular representation, as it aggregates information from the entire sequence through successive attention layers. Alternative pooling strategies, including mean pooling over valid tokens and aggregation across multiple final layers, were evaluated in ablation studies but are not part of the primary architecture. The pooled molecular embedding was passed to a regression head consisting of a linear projection layer that maps the transformer embedding to a single scalar output representing the predicted log_10_ LD_50_ value. Dropout regularization was applied prior to the regression layer to mitigate overfitting. The [CLS] pooling strategy was selected as the default molecular embedding because successive attention layers propagate contextual information from all tokens into this representation, effectively forming a learned global summary vector. This design allows the model to determine which structural features are most predictive of toxicity rather than relying on predefined aggregation rules.

A weighted regression loss function was employed to account for experimental uncertainty and replicate variability. Samples associated with higher uncertainty were assigned lower weights, reducing their influence on parameter updates and improving robustness to noisy labels. Incorporating uncertainty-weighted loss was motivated by the observation that experimental LD_50_ measurements often exhibit inter-study variability due to species differences, assay conditions, and reporting uncertainty. Assigning lower weights to noisy observations reduces gradient variance and prevents the model from overfitting to unreliable measurements, effectively implementing a form of heteroscedastic noise modeling within the optimization process. To prevent overfitting, multiple regularization strategies were applied including dropout within the regression head, weight decay on all trainable parameters and early stopping based on validation mean absolute error (MAE). MAE was calculated using Equation 1. Training was terminated when validation MAE failed to improve for a predefined number of epochs. Model performance was assessed using multiple regression metrics, including MAE, root mean squared error (RMSE), and coefficient of determination (R²), computed on held-out validation and test sets. MAE was used as the primary optimization and early-stopping criterion due to its robustness to outliers and interpret-ability in toxicity prediction tasks. For classification experiments, model performance was evaluated using class-wise precision metrics on the held-out external test set. Precision for each class was calculated using Equation 2, computed separately for the four LD_50_ toxicity categories. Class 0, 1, 2, 3 indicated molecules falling below range or equal to 50, 500, 5000 and more than 5000 respectively.

(1) $$MAE=\frac{1}{n}\sum_{i=1}^{n}\left|y_{i}-{\hat{y}}_{i}\right|$$

(2) $$Precision=\frac{TruePositive}{\left(TruePositive+FalsePositive\right)}$$

### Reproducibility and implementation details

All experiments were conducted using Python version 3.10 framework to ensure reproducibility and transparency. Molecular preprocessing and SMILES canonicalization were performed using RDKit versoin 2024.3. Model implementation and training were carried out using PyTorch 2.7.1 and the Hugging Face Transformers library 4.57 (36). Hyperparameter optimization was performed using Optuna version 4.4. Training and evaluation were executed on Linux-based Ubuntu operating system, equipped with NVIDIA GPU with CUDA version 12.4 (37). Experiments were performed on a single GPU both for pretraining as well as fine tuning for the regression task. For reproducibility, random number were generated and initialized with seed 42. Training, validation, and test datasets were generated from the main data in the ratio of 8:1:1 respectively and reused across all models to guarantee consistent comparisons using the Scikit learn package (38). Model checkpoints were saved during training, based on the validation MAE and was used for final external test validation. Pretrained as well as fine-tuned model weights were stored in a safe and portable format (.safetensors), along with tokenizer configurations, which can assist in exact reproduction of reported results and facilitate the downstream reuse.

### Comparative modeling studies

To contextualize the performance of the proposed ModernBERT-based LD_50_ predictor, we conducted systematic comparisons with three widely adopted molecular modeling paradigms: ChemProp (39), ChemBERT and ensemble learning. We included two tree based ensemble regression models namely Gradient Boosting Regressor (GBR) and Histogram based Gradient Boosting Regressor (HGB), for prediction (40–43). An ensemble model was constructed by linearly combining predictions from GBR and HGB. All models were trained exclusively on the training set, while the internal validation set was used for model selection and ensemble optimization. The optimal ensemble weight was determined by minimizing the MAE on the internal validation set, after which the optimized ensemble was held fixed and evaluated on the external test set. These models were selected as representative baselines due to their extensive use in computational toxicology and molecular property prediction. ChemProp is a state-of-the-art directed message passing neural network (D-MPNN) that operates directly on molecular graphs. It was the top method which was peer reviewed and reported in ADMET benchmark scoreboard with minimal MAE. In this framework, atoms are treated as nodes and bonds as directed edges, with information propagated along bonds to learn chemically meaningful representations. ChemProp has demonstrated strong performance across a wide range of QSAR and ADMET benchmarks and is commonly regarded as a competitive baseline for toxicity prediction tasks. In this study, ChemProp models were trained using canonical molecular graph representations derived from SMILES strings. While message-passing networks encode explicit chemical connectivity, their locality bias can restrict representation of distal interactions unless many propagation steps are used, which may lead to oversmoothing or optimization instability. Transformer attention mechanisms provide an alternative inductive bias that directly models global interactions, potentially offering an advantage for endpoints such as toxicity that depend on distributed structural effects. ChemBERT represents an earlier generation of SMILES-based transformer models, pretrained using masked language modeling on large molecular corpora (44). By modeling SMILES as a sequence, ChemBERT enables learning of contextual token representations without requiring explicit graph construction. As a result, ChemBERT has been extensively adopted as a baseline in molecular property prediction studies, including toxicity modeling. We have used DeepChem platform for evaluating ChemBERT in our studies (45). The ChemModernBERT SMILES model employed in this work builds upon the strengths of SMILES-based language modeling while addressing several limitations of earlier approaches. Compared to ChemBERT, it benefits from deeper architectures, improved attention mechanisms, and large-scale curriculum-based pretraining on millions of SMILES strings. Relative to ChemProp, it avoids reliance on handcrafted graph representations and enables end-to-end learning directly from molecular sequences. By evaluating all three approaches under identical data splits, preprocessing pipelines, and evaluation protocols, this study provides a fair and systematic comparison between graph-based models (ChemProp), early SMILES transformers (ChemBERT), and modern large-scale molecular language models (ChemModernBERT) for LD_50_ prediction. The performance gains observed for ChemModernBERT are therefore hypothesized to arise from the combination of factors namely, large-scale chemical pretraining enabling transferable representations, deep contextual attention allowing global structural reasoning, and curriculum-induced invariance to SMILES permutations. These properties collectively increase representation expressivity while maintaining generalization across chemically diverse compounds.

## Results and discussion

### Dataset collection and preprocessing

**Figure 1:**
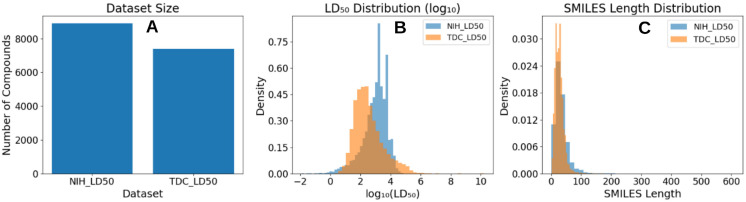
**(A)** Comparison of dataset sizes, showing the number of compounds in the curated NIH LD_50_ dataset and the TDC LD_50_ benchmark. **(B)** Distribution of LD_50_ values plotted on a log_10_ scale, highlighting differences in toxicity range and spread between the two datasets. **(C)** Distribution of SMILES sequence lengths, illustrating structural complexity and variability across compounds.

**Figure 2:**
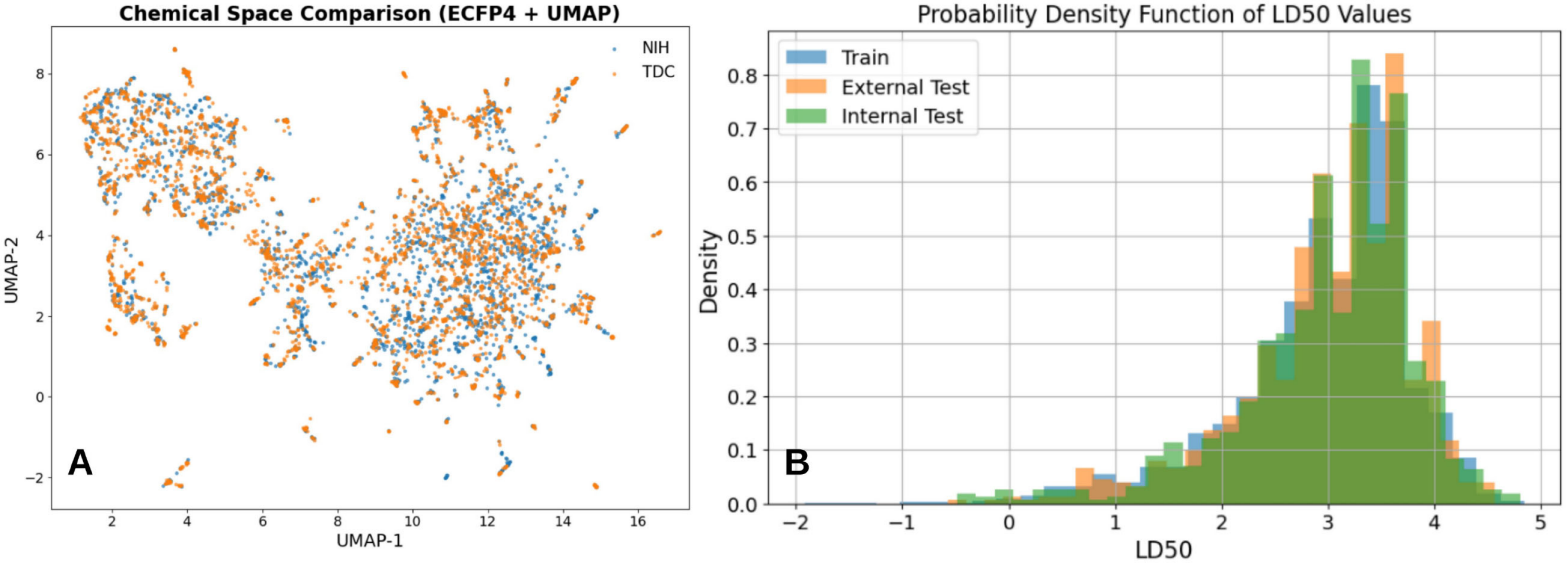
**(A)** UMAP projection of ECFP4 fingerprints comparing chemical space coverage of NIH and TDC LD_50_ datasets, showing extensive overlap with additional unique regions captured by the NIH dataset. **(B)** Distribution of train, internal test and external test dataset based on the LD_50_ values on log scale.

Systematic dataset curation and preprocessing ensured a chemically consistent and reproducible benchmark suitable for modern molecular language models. Experimental acute oral LD_50_ records were accessed from publicly available toxicological databases maintained by the U.S. National Library of Medicine and the U.S. Environmental Protection Agency on 6 September 2025. RDKit-based SMILES parsing and canonicalization removed invalid or unparseable entries and eliminated redundant representations arising from alternative SMILES encodings, enforcing a one-to-one correspondence between molecular graphs and training samples. Filtering for experimentally measured LD_50_ values expressed exclusively in mg/kg reduced unit ambiguity, while log_10_ transformation and robust aggregation of multiple measurements per compound mitigated skewness and experimental variability. Importantly, molecules containing heavy atoms or metal centers were retained when structurally parsable, extending chemical coverage beyond that of many descriptor-based pipelines. Collectively, the increased dataset scale, higher scaffold diversity, and rigorous preprocessing establish a strong foundation for fine-tuning pretrained transformer architectures and enable more realistic evaluation of LD_50_ prediction performance in regulatory and environmental toxicology settings. The curated NIH LD_50_ dataset comprises 8,898 unique compounds, exceeding the size of the widely used Therapeutics Data Commons (TDC) LD_50_ benchmark (46), which contains 7,385 compounds, and providing substantially broader chemical coverage (**Figure 1A**). It has more balanced distribution of toxicity values (**Figure 1B**) and larger maximum sequence length (**Figure 1C**). Detailed comparison has been summarized in supplementary section S2. Characteristics of NIH dataset indicated improved coverage of both chemical space and toxicity extremes, which is visually confirmed by the broader and more dispersed distribution of NIH compounds in the UMAP projection (**Figure 2A**). Higher scaffold diversity is critical for robust generalization, as it mitigates overfitting to recurrent chemotypes and enables models to learn toxicity-relevant patterns that transfer across structurally distinct compounds. For transformer-based SMILES models, exposure to diverse scaffold topologies and syntactic realizations further strengthens the model’s ability to capture long-range dependencies, branching patterns, and ring systems that are central to chemical reasoning. Also, using the Murco scaffold based separation of train, internal test and external test using the stratified separation, we were able to get uniformly distributed train, internal and external test sets for ML modeling (**Figure 2B**). Consequently, the NIH dataset provides a more challenging and informative training environment than smaller benchmark collections. A summary of characteristics of the current NIH dataset are summarized in **Table 1**.

**Table 1:** Numerical comparison with TDC LD_50_ benchmark.

Characteristic	Curated LD_50_ dataset (this study)	TDC LD_50_ benchmark
Number of Unique compounds	**8,898**	7,385
Data sources	NLM, EPA (primary toxicological repositories)	Aggregated benchmark
Endpoint	Acute oral LD_50_	Acute oral LD_50_
Units	mg/kg (standardized)	mg/kg
Target transformation	log_10_ LD_50_	log_10_ LD_50_
Molecules with multiple measurements	Aggregated using robust statistics	Typically single-value
SMILES processing	RDKit canonicalization	Pre-standardized
Heavy atom/metal-containing molecules	Retained if parsable	Often excluded

Bold values indicate larger dataset.

### Pretraining ModernBERT

**Figure 3:**
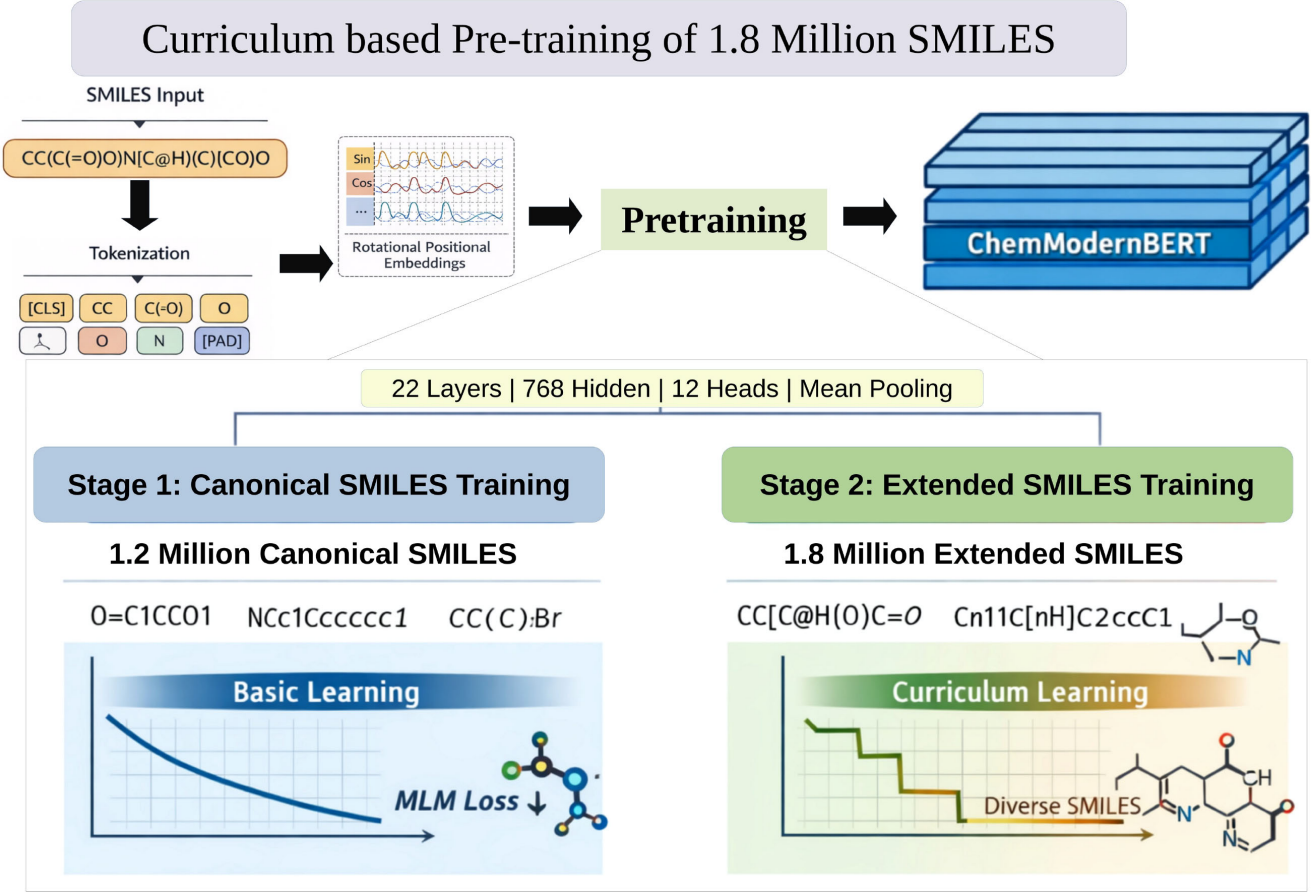
Curriculum-based two-stage pretraining of ChemModernBERT on 1.8 million SMILES, progressing from canonical to structurally diverse sequences to improve representation learning. The “sin” and “cos” labels denote trigonometric components used in RoPE, which encode relative positional information in tokenized SMILES sequences.

We pretrained a ModernBERT-based masked language model tailored for molecular SMILES representations using a two-stage curriculum learning strategy. The whole workflow has been summarized in **Figure 3**. In **Figure 3**, the sinusoidal sin and cos annotations visualize the mathematical basis of Rotational Positional Embeddings (RoPE) encoding rather than training dynamics. They represent relative token positions. The model follows the ModernBertForMaskedLM architecture with 22 transformer layers, a hidden size of 768, and 12 attention heads, providing representational capacity comparable to BERT-base while incorporating architectural refinements optimized for sequence efficiency and chemical syntax modeling. A vocabulary of 50,368 tokens, including chemically meaningful special tokens ([CLS], [SEP], [MASK], [PAD], [UNK]), was used consistently across pretraining and downstream tasks. The maximum positional embedding length was set to 160 tokens, reflecting the typical length distribution of canonical and extended SMILES strings while minimizing unnecessary padding.

**Figure 4:**
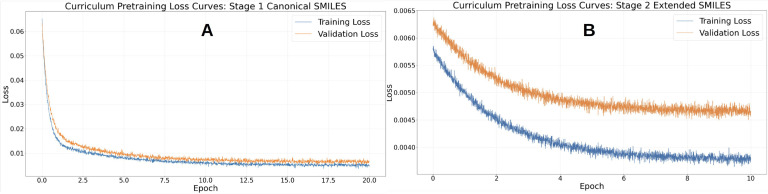
**(A)** Stage 1 (canonical SMILES): training and validation masked language modeling (MLM) losses over 20 epochs showing smooth convergence and stable optimization on standardized molecular representations. **(B)** Stage 2 (extended SMILES): losses over 10 epochs demonstrating rapid stabilization after introduction of structurally diverse SMILES encodings, indicating effective knowledge transfer and improved robustness to representational variability.

Training was conducted for a total of 30 epochs following a curriculum learning paradigm that progressively increased structural complexity in the training data. In the first stage, the model was trained for 20 epochs on approximately 1.1 million canonicalized SMILES strings. This phase enabled the model to learn fundamental SMILES grammar, common functional groups, and local syntactic dependencies under low representational variability. During this stage, masked language modeling (MLM) loss decreased smoothly throughout training, indicating stable convergence and effective acquisition of core chemical language patterns (**Figure 4a**). Following convergence on canonical SMILES, the model was further trained for 10 epochs on an expanded dataset of approximately 1.8 million extended SMILES strings (**Figure 4b**). Final MLM loss for validation dataset has been summarized in **Supplementary Table 1** of Section S3. These representations introduced alternative atom orderings, stereochemical annotations, and increased syntactic diversity while preserving chemical validity. This second stage improved robustness to non-canonical SMILES representations, enhanced sensitivity to stereochemical markers, and strengthened the model’s ability to capture long-range dependencies across branched and cyclic molecular structures, without inducing optimization instability or catastrophic forgetting.

Across all training epochs, optimization remained stable without the need for gradient checkpointing or aggressive regularization, suggesting that the curriculum strategy contributed to implicit regularization during pretraining. The final pretrained model was saved in model.safetensors format along with the complete tokenizer configuration, enabling safe reuse and seamless integration into downstream fine-tuning pipelines. Intermediate checkpoints further allow training to be resumed or extended if required, although loss convergence indicated no immediate need for additional pretraining. Overall, curriculum-based pretraining yielded chemically informed and robust SMILES embeddings well suited for downstream molecular property prediction tasks, including LD_50_ regression, ADMET modeling, and toxicity classification. Compared with models trained solely on canonical SMILES, the curriculum-trained representations are expected to generalize more effectively to real-world settings where molecules may appear in diverse syntactic forms. While graph-based models explicitly encode molecular topology, the present approach offers greater scalability and ease of integration with other sequence-based or multimodal architectures while still capturing chemically meaningful structure through domain-specific tokenization and training.

### Model training for LD_50_ prediction

**Figure 5:**
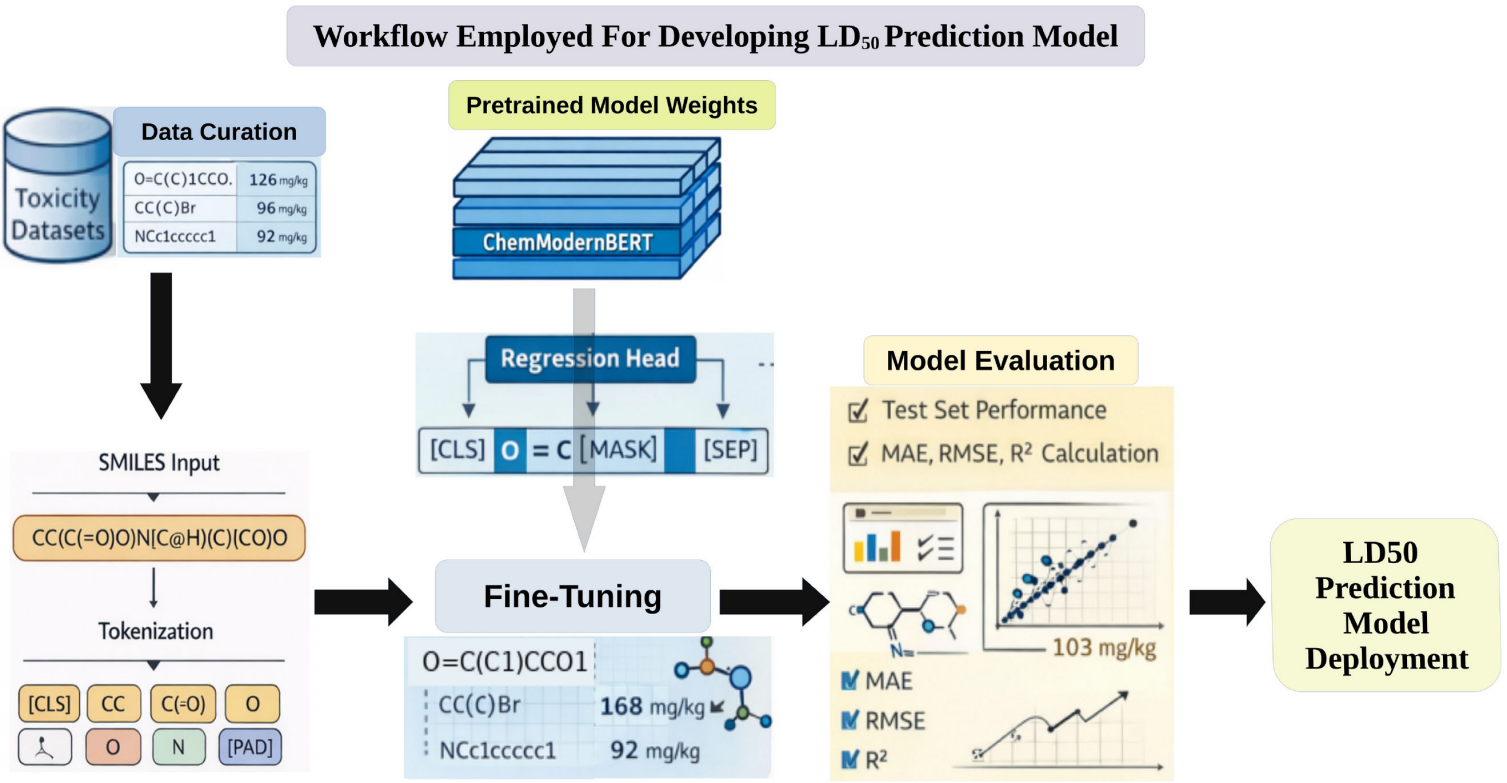
Workflow illustrating data curation, SMILES tokenization, ChemModernBERT fine-tuning, evaluation, and deployment for LD_50_ prediction.

The proposed ChemModernBERT SMILES model demonstrates strong predictive performance for LD_50_ estimation across training, validation, and held-out test sets. By fine-tuning a large pretrained molecular language model, the approach effectively captures both syntactic and semantic information embedded within SMILES representations. A schematic overview of the complete LD_50_ prediction workflow is provided in **Figure 5**, summarizing data curation, tokenization, model fine-tuning, evaluation, and deployment. Evaluation using multiple regression metrics including MAE, RMSE, and R² indicates that the model achieves robust generalization, with minimal performance degradation between validation and test splits. The use of log_10_ transformed LD_50_ values further stabilizes optimization and reduces the influence of extreme toxicity values, enabling the model to learn smoother functional mappings between molecular structure and toxicity response. A key strength of the proposed architecture lies in its transformer encoder pretrained on large-scale molecular corpora. During pretraining, the model learns chemically informative contextual embeddings that capture local atomic environments, long-range intramolecular dependencies, and scaffold-level structural patterns. Fine-tuning these representations for LD_50_ regression enables the model to reuse pretrained chemical knowledge rather than relying solely on task-specific supervision. In contrast to traditional descriptor or fingerprint based regression models, the transformer-based approach operates directly on raw SMILES strings, eliminating the need for features. This end-to-end learning paradigm allows the model to automatically discover task-relevant structural motifs associated with acute toxicity. An additional advantage is the ability of SMILES-based language models to accommodate heavy-atom and metal-containing compounds, which are frequently excluded or difficult to process in descriptor-based pipelines due to limitations in feature computation and molecular sanitization. The incorporation of RoPE plays a critical role in modeling molecular sequences (47). Unlike absolute positional embeddings, RoPE encodes relative positional information through sinusoidal rotations applied within the attention mechanism. This design enables the self-attention layers to better capture relative token distances, which is particularly important for SMILES strings where chemically meaningful relationships often depend on relative rather than absolute positions. In the context of molecular sequences, RoPE facilitates improved representation of ring closures, branching patterns, and long-range dependencies between distant atoms. Qualitatively, this contributes to more stable attention patterns and enhances the model’s ability to generalize across molecules of varying length and complexity.

For LD_50_ prediction, a pooled molecular representation is required to map token-level embeddings to a single scalar output. The default configuration employs the embedding of the special [CLS] token from the final transformer layer, which aggregates information from the entire sequence via self-attention. Ablation studies comparing alternative pooling strategies such as mean pooling across tokens and pooling over multiple final layers revealed that [CLS]-based pooling consistently provided competitive or superior performance. This suggests that the pretrained model effectively learns to concentrate global molecular information within the [CLS] token during fine-tuning, making it a suitable summary representation for regression tasks. The regression head consists of a lightweight linear projection applied to the pooled molecular embedding. Despite its simplicity, this design proved sufficient when coupled with a powerful pretrained encoder. Dropout regularization was applied prior to the regression layer to mitigate overfitting, particularly given the limited size of labeled toxicity datasets relative to pretraining corpora. Fine-tuning all layers of the model yielded improved performance compared to freezing the encoder, indicating that task-specific adaptation of the molecular representations is beneficial for LD_50_ prediction. However, freezing early layers while fine-tuning higher layers produced only marginal performance degradation, highlighting the transferability of lower-level chemical features learned during pretraining. Experimental LD_50_ measurements often exhibit variability due to differences in experimental conditions, species, and assay protocols. To address this, a weighted regression loss was employed, reducing the influence of noisy or weakly constrained labels during training. This approach led to improved validation stability and reduced sensitivity to outliers, as reflected by lower MAE and RMSE values. The weighted loss formulation encourages the model to focus on well-supported measurements, resulting in more reliable toxicity predictions and improved robustness when evaluated on unseen data.

### Comparison with traditional approaches

**Figure 6:**
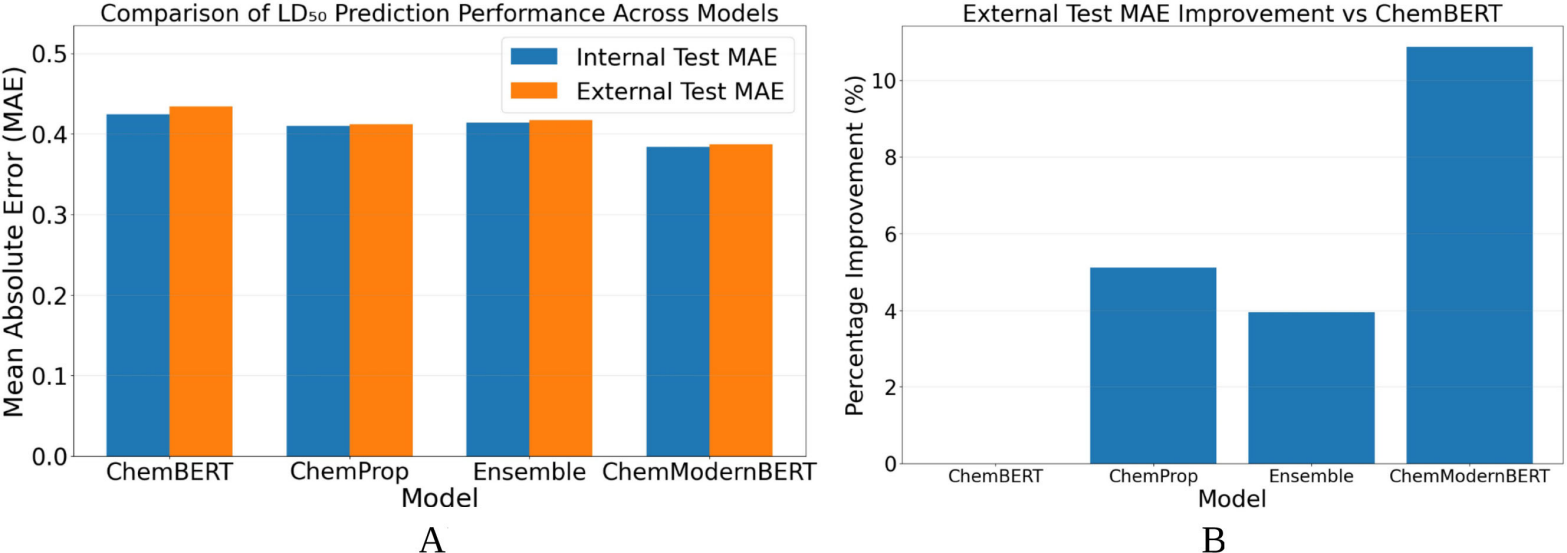
**(A)** Internal and external MAE comparison across models. **(B)** External test MAE improvement relative to ChemBERT.

**Figure 7:**
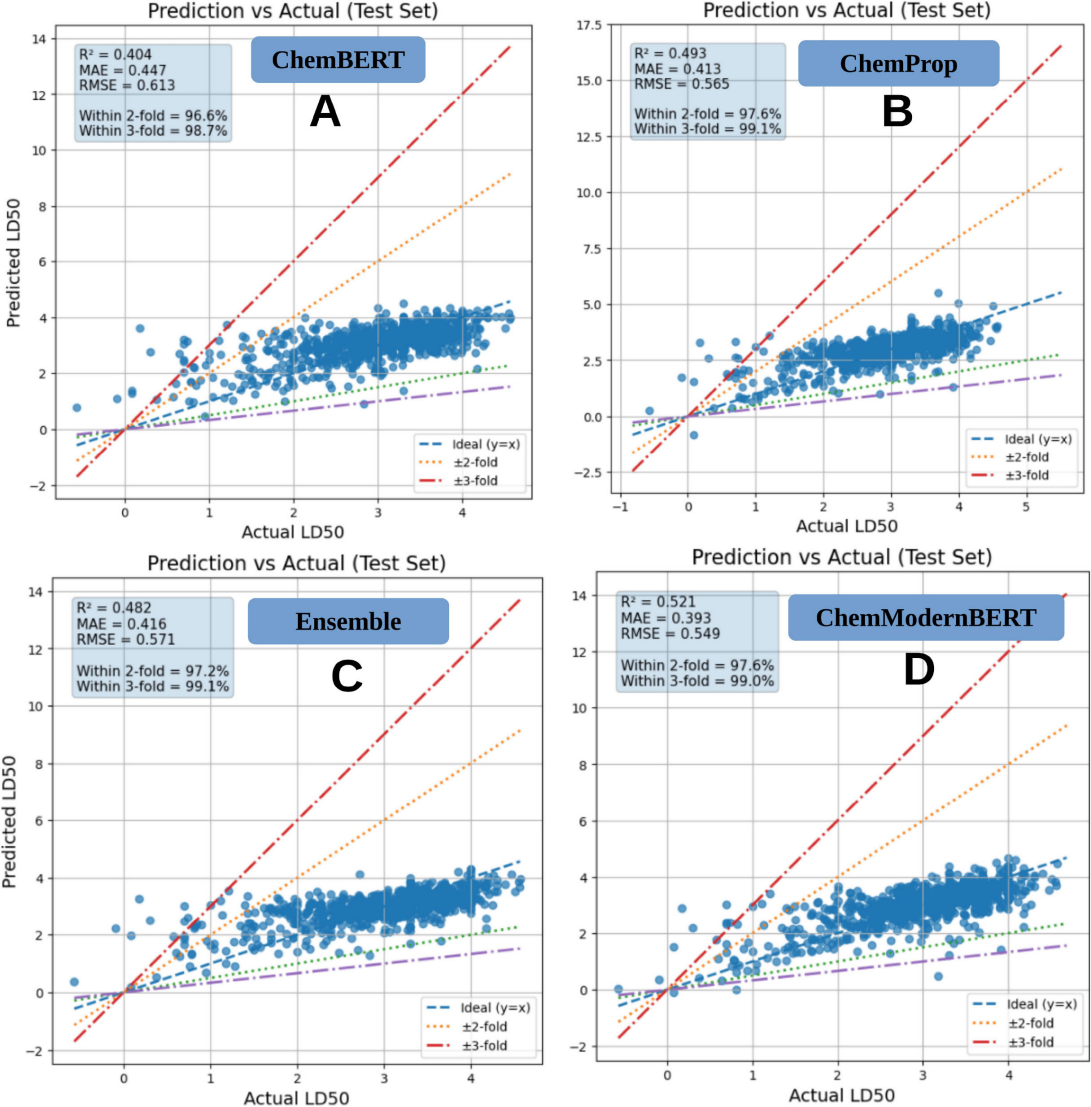
**(A)** R^2^, MAE, RMSE values of ChemBERT based model. **(B)** R^2^, MAE, RMSE values of ChemProp based model. **(C)** R^2^, MAE, RMSE values of Ensemble model. **(D)** R^2^, MAE, RMSE values of ChemModernBERT based model. Dashed lines indicate the ideal y = x relationship and ±2-fold and ±3-fold error bounds.

While direct numerical comparisons depend on dataset composition and data-splitting strategies, the ModernBERT–SMILES framework overcomes several inherent limitations of traditional QSAR approaches. Classical models based on fixed fingerprints or handcrafted descriptors rely on predefined chemical features, which often fail to capture subtle structural variations and long-range intramolecular dependencies in complex molecules. In contrast, the transformer encoder learns adaptive, context-aware molecular representations directly from raw SMILES sequences, enabling more expressive modeling of chemical syntax and structural context. As summarized in **Table 2** and visualized in **Figures 6A**, ChemModernBERT achieves the lowest mean absolute error (MAE) on both internal and external test sets, demonstrating superior predictive accuracy compared with ChemBERT, Ensemble model and ChemProp. Importantly, the generalization gap between internal and external performance remains small across all evaluated models, with ChemModernBERT exhibiting a gap of only +0.003. This indicates that the observed performance gains are not driven by overfitting but rather by improved representation learning and effective transfer of pretrained chemical knowledge. Quantitatively, ChemModernBERT achieves a 9.45% reduction in external MAE relative to ChemBERT (**Figure 6B**), highlighting the substantial benefit of large-scale molecular language model pretraining. ChemProp also improves performance, yielding a 4.84% reduction, but to a lesser extent. Ensemble model had better results in MAE having 3.92% reduction. Together, these results underscore the advantage of pretrained transformer-based molecular models as scalable and general-purpose backbones for toxicity prediction, offering improved generalization compared with both classical QSAR methods and task-specific architectures. Detailed performance comparisons are summarized in **Table 3**, showing progressive improvement from ChemBERT to ChemProp and ChemModernBERT. ChemModernBERT achieves the best accuracy (R² = 0.521, MAE = 0.393, RMSE = 0.549) with the tightest clustering around the ideal prediction line. All models show strong robustness, with over 96% and 99% of predictions falling within ±2-fold and ±3-fold error bounds, respectively (**Figure 7**).

**Table 2:** The table summarizes the MAE values of the different methods on the identical internal and external test set.

Sr. No.	Name of model	Internal test MAE	External test MAE	Generalization gap	% Improvement (external)
1.	ChemBERT	0.424	0.434	+0.010	–
2.	ChemProp	0.410	0.413	+0.003	4.84%
3.	Ensemble Model	0.414	0.417	+0.003	3.92%
4.	ChemModernBERT	0.390	0.393	+0.003	**9.45%**

Bold value indicate the highest improvement among all the models.

**Table 3:** The table summarizes the R^2,^ MAE, RMSE, within 2 fold and within 3 fold values of the different methods on the identical external test set.

Sr. No.	Name of model	External test MAE	External test RMSE	External test R_2_	Within 2 fold (%)	Within 3 fold (%)
1.	ChemBERT	0.447	0.613	0.404	96.6	98.7
2.	ChemProp	0.413	0.565	0.493	**97.6**	**99.1**
3.	Ensemble	0.417	0.571	0.482	97.2	**99.1**
4.	ChemModernBERT	**0.393**	**0.549**	**0.521**	**97.6**	99.0

The bold value indicate the best value achieved in terms of MAE, RMSE, R2, Within 2 fold and Within 3 fold. 2 bold values indicate identical performance in that metrics.

Overall, these findings support the use of pretrained molecular language models as strong, general-purpose backbones for toxicity prediction. The ModernBERT based approach enables end-to-end learning directly from molecular sequences without reliance on handcrafted descriptors. By leveraging self-attention mechanisms to capture complex structural patterns and chemical context, the model generalizes effectively across diverse chemical scaffolds and functional groups. This capability is particularly valuable for toxicity modeling, where subtle structural variations can result in substantial changes in biological response. Compared with task-specific QSAR regressors and graph-based networks, transformer-based SMILES models offer improved scalability, reduced dependence on manual feature engineering, and robust generalization across independent test sets. To further evaluate model reliability beyond aggregate performance metrics, we analyzed class-wise precision across toxicity categories. This analysis revealed that traditional approaches exhibited higher variability between classes, suggesting sensitivity to class imbalance and chemical distribution shifts. In contrast, the proposed model maintained consistently strong performance across multiple toxicity ranges, indicating improved stability of learned molecular representations. The ensemble baseline achieved the most uniform class performance, supporting the hypothesis that combining heterogeneous predictors can mitigate class-specific bias. These results, summarized in **Table 4**, highlight that modern molecular language models provide not only competitive overall accuracy but also more reliable behavior across chemically diverse toxicity categories. From a cancer risk assessment perspective, improved LD_50_ prediction enables more reliable prioritization of compounds for downstream carcinogenicity testing and dose-range selection.

**Table 4:** Class-wise precision comparison across toxicity categories for all evaluated models.

Sr. No.	Name of model	Precision			
		Class 0	Class 1	Class 2	Class 3
1.	ChemBERT	0.52	0.44	0.68	0.36
2.	ChemProp	0.52	0.49	0.70	0.42
3.	Ensemble	0.64	0.52	0.65	0.68
4.	ChemModernBERT	0.56	0.47	0.71	0.53

Class 0, 1, 2, 3 indicates molecules falling below range or equal to 50, 500, 5000 and more than 5000 respectively. Precision for each class was computed separately for the four LD_50_ toxicity categories using Equation 2.

### Limitations and future directions

Currently, the base model displayed strong performance compared to the graph based chemProp model and LLM based ChemBERT, but it suffers from limitations. First, SMILES-based representations remain sensitive to sequence canonicalization and tokenization choices. The robustness of the model can be further improved by creating fusion based models having graph data as well as transformer layer, and hyperparameter tuning can improve it further. The regression head currently used was simple and by adding more expressive heads or multitask learning with related toxicity endpoints may improve results. Future work may explore hybrid architectures that combine transformer-based sequence embeddings with graph neural network representations or physicochemical descriptors, as well as uncertainty-aware prediction frameworks to better quantify confidence in LD_50_ estimates. Second, the training dataset may contain inherent biases that affect generalization. Public toxicity datasets often exhibit uneven chemical space coverage, overrepresentation of certain scaffolds, and variability in experimental protocols. Such factors can lead to distributional shifts between training and real-world deployment environments. Future work should therefore evaluate domain applicability boundaries, perform scaffold-level robustness analyses, and incorporate curated external datasets representing broader chemical diversity. Third, the present model is optimized specifically for prediction of acute oral LD_50_ and should not be interpreted as a surrogate for chronic toxicity, carcinogenic potential, or long-term biological risk. While acute toxicity is an important component of chemical safety evaluation, cancer-relevant risk assessment depends on additional mechanistic, metabolic, and exposure-duration factors that lie beyond the scope of this model. Integrating multimodal toxicological endpoints or adopting multitask learning strategies may enable more comprehensive hazard profiling. Although the observed R² values are moderate, they fall within the range commonly reported for data-driven toxicity prediction models trained on heterogeneous experimental datasets. It is important to emphasize that computational toxicity predictions were intended to support and not replace the experimental evaluation or regulatory judgment. Over-reliance on predicted LD_50_ values without considering experimental validation, exposure context, or mechanistic evidence could lead to inappropriate risk interpretation. Accordingly, models such as the one presented here were best deployed as prioritization tools within integrated assessment pipelines, where predictions guide experimental design, dose selection, and compound triage rather than serving as standalone decision criteria. Future research will focus on improving robustness, interpretability, and applicability through hybrid sequence-graph architectures, expanded pretraining corpora, cross-domain validation, and integration with physiologically based pharmacokinetic and systems toxicology frameworks. These directions aim to ensure that advances in molecular language modeling translate into reliable, transparent, and responsibly deployable tools for chemical safety assessment.

## Conclusion

This study demonstrates that fine-tuned regression-based LD_50_ prediction using pretrained transformer-based molecular language models provides an effective and flexible modeling framework for toxicity assessment. In particular, modern SMILES-based transformer architectures incorporating enhanced positional encoding and deep contextual representation learning consistently outperform both earlier transformer variants and established graph-based message-passing networks. The results in current studies suggest that representation learning scale, rather than architecture specialization alone, may be a key driver of toxicity prediction performance. The robust and reproducible performance of ChemModernBERT across internal and external test sets highlights its superior ability to capture toxicity-relevant molecular features, resulting in improved generalization across chemically diverse compounds. Importantly, these performance gains are achieved on an expanded and chemically diverse dataset that better reflects real-world chemical space than conventional benchmarks. Such diversity is critical for regulatory toxicology and cancer risk assessment, where model robustness and generalizability are essential. Class-level evaluation further demonstrates that predictive performance is not uniform across toxicity ranges, emphasizing the importance of reporting stratified metrics when assessing reliability in computational toxicology. This analysis indicates that modern molecular language models not only achieve strong overall accuracy but also maintain stable behavior across multiple toxicity categories, supporting their suitability for chemically heterogeneous screening scenarios. Moreover, ChemModernBERT exhibits favorable scaling behavior, suggesting that modern molecular language models benefit more substantially from increased data availability than traditional graph-based approaches, an advantage likely to become increasingly pronounced as toxicological datasets continue to expand in size and structural complexity. Beyond serving as a standalone predictor, the pretrained ChemModernBERT encoder provides a versatile foundation for integration into hybrid modeling frameworks. Its learned molecular representations can be readily combined with graph neural networks or physicochemical descriptors, enabling the development of multimodal fusion models that further enhance predictive accuracy and robustness. Notably, the curriculum-pretrained ChemModernBERT introduced in this work represents a reusable and extensible backbone for future toxicological modeling, including large-scale carcinogenicity prediction and comprehensive chemical safety assessment. Collectively, these findings support the adoption of modern molecular language models as high-performing predictors and transferable representation backbones capable of enabling accurate, scalable LD_50_ prediction, thereby advancing large-scale chemical safety screening, strengthening cancer-relevant toxicity assessment, and facilitating integration into evidence-based early-stage risk assessment and regulatory decision-making pipelines. Future research will explore hybrid sequence–graph architectures, expanded pretraining datasets, and uncertainty-aware prediction strategies to improve robustness and applicability. Integration with systems toxicology frameworks may further enable practical deployment of molecular language models for real-world chemical safety assessment.

## Data Availability

The original contributions presented in the study are included in the article/Supplementary Material. The code and related resources used in this study are available at the following GitHub repository: [https://github.com/tanush84/Machine-Learning-Driven-LD-Prediction-Using-Modern-Molecular-Language-Models]. Further inquiries can be directed to the corresponding author.
