# Supplementary material for: Machine learning driven LD50 prediction for cancer risk assessment using modern molecular language models
Source: Front Oncol. 2026 Mar 19;16:1788240. doi: 10.3389/fonc.2026.1788240 (PMC13043998; doi:10.3389/fonc.2026.1788240)
Supplement: Supplementary file 1 [file Table1.docx]

**Supplementary Data:**

**Section S1: Preprocessing Script.**

## Preprocessing Script.

from rdkit import Chem

from rdkit.Chem import rdMolStandardize

def standardize_smiles(smiles):

try:

# Parse SMILES

mol = Chem.MolFromSmiles(smiles)

if mol is None:

return None

# Sanitize molecule

Chem.SanitizeMol(mol)

# ---- Standardization ----

# Remove salts / keep largest fragment

mol = rdMolStandardize.FragmentParent(mol)

# Normalize functional groups

normalizer = rdMolStandardize.Normalize()

mol = normalizer.normalize(mol)

# Neutralize charges

uncharger = rdMolStandardize.Uncharger()

mol = uncharger.uncharge(mol)

# Tautomer canonicalization

te = rdMolStandardize.TautomerEnumerator()

mol = te.Canonicalize(mol)

# Remove explicit hydrogens

mol = Chem.RemoveHs(mol)

# Final canonical SMILES

smiles_std = Chem.MolToSmiles(mol, canonical=True)

# Final validation

if Chem.MolFromSmiles(smiles_std) is None:

return None

return smiles_std

except:

return None

# Example usage

if __name__ == "__main__":

test_smiles = [

"CC(=O)[O-].[Na+]",

"C1=CC=CN=C1",

"invalid_smiles"

]

for smi in test_smiles:

print(smi, "→", standardize_smiles(smi))

**Section S2: Comparison with TDC dataset:**

**The NIH dataset spans a wider toxicity range, particularly at the highly toxic end, with log₁₀(LD₅₀) values extending to −1.92 compared with 0.29 for TDC. While both datasets exhibit comparable central tendencies, the NIH dataset shows a slightly higher mean log₁₀(LD₅₀) (2.98 vs. 2.54) and a marginally lower standard deviation, suggesting a more balanced distribution of toxicity values (Figure 1B). In addition, NIH compounds display greater structural complexity, as reflected by longer and more variable SMILES representations, with a higher average SMILES length (34.3 vs. 27.5) and a substantially larger maximum sequence length (608 vs. 174) (Figure 1C). Together, these characteristics indicate improved coverage of both chemical space and toxicity extremes, which is particularly advantageous for training and fine-tuning transformer-based molecular language models. Beyond compound count alone, the NIH dataset exhibits markedly higher scaffold diversity, containing 2,493 unique Bemis–Murcko scaffolds compared with 1,677 scaffolds in the TDC LD₅₀ benchmark. This expanded scaffold repertoire indicates broader representation of core molecular frameworks and reduced redundancy across structural motifs.**

Section S3:

Supplementary Table S1: The table indicates the MLM loss observed after the Canonical and Extended

Canonical training protocol stages on the validation dataset.

| Stage | Dataset Size | Epochs | Final MLM Loss |
| --- | --- | --- | --- |
| Canonical | 1.1M | 20 | 0.00632 |
| Extended | 1.8M | 10 | 0.00465 |
